# Bioactivity-Guided Fractionation and Mechanistic Insights into *Aristolochia ringens* Root Extract-Induced G_1_ Phase Arrest and Mitochondria-Mediated Apoptosis in Human Colon Adenocarcinoma Cells

**DOI:** 10.3390/ph18091250

**Published:** 2025-08-23

**Authors:** Saheed O. Anifowose, Abdalrhaman M. Salih, Musa K. Oladejo, Ahmad Rady, Mobarak S. Al Mosallam, Hasan A. Aljohi, Mansour I. Almansour, Saad Hussin Alkahtani, Ibrahim O. Alanazi, Badr A. Al-Dahmash

**Affiliations:** 1 Zoology Department, College of Science, King Saud University, P.O. Box 2455, Riyadh 11451, Saudi Arabia; olaidesaeed@hotmail.com (S.O.A.); 446110029@student.ksu.edu.sa (M.K.O.); ahabdo@ksu.edu.sa (A.R.);; 2 Botany and Microbiology Department, College of Science, King Saud University, P.O. Box 2455, Riyadh 11451, Saudi Arabia; abdalrahamanm@gmail.com; 3 Healthy Aging Research Institute, Health Sector, King Abdulaziz City for Science and Technology, Riyadh 11442, Saudi Arabia; 4 Applied Genetics Technology Institute, King Abdulaziz City for Science and Technology, Riyadh 11442, Saudi Arabia

**Keywords:** bioactive phytochemicals, anticancer secondary metabolites, *Aristolochia ringens*, apoptosis, mitochondria membrane depolarizer

## Abstract

**Background/Objectives**: *Aristolochia ringens*, a medicinal plant widely used in traditional medicine, has shown potential therapeutic applications. This study aimed to investigate the anticancer mechanism of action of its crude extract against human colorectal adenocarcinoma cells (Caco-2 and HT-29). **Methods**: Cell viability was assessed using the MTT assay to determine IC_50_ values. Immunofluorescence microscopy was used to examine nuclear morphology and microtubule integrity. Flow cytometry with PI staining was used for cell cycle analysis and Annexin V-FITC/PI staining for apoptosis detection. Mitochondrial membrane potential was evaluated using JC-1 dye. Bioactivity-guided fractionation was performed via HPLC, and GC–MS was used to profile active constituents. **Results**: The extract exhibited dose-dependent cytotoxicity with IC_50_ values below 30 µg/mL in colon adenocarcinoma cell lines. Treated Caco-2 cells showed nuclear shrinkage and disrupted microtubules. PI-based flow cytometry revealed G_1_ phase arrest, and Annexin V-FITC/PI staining indicated enhanced late apoptosis. JC-1 staining demonstrated mitochondrial depolarization. HPLC fractionation identified fractions 2 and 3 as active, and preliminary GC–MS analysis tentatively annotated the presence of alkaloids, sesquiterpenes/diterpenes, and steroidal compounds. **Conclusions**: *A. ringens* exerts anticancer effects through a mitochondria-mediated apoptotic pathway, involving G_1_ checkpoint arrest and cytoskeletal disruption. These findings provide the first integrated cellular and mechanistic evidence of its anticancer potential in colorectal cancer, supporting its promise as a source of novel therapeutic lead compounds.

## 1. Introduction

Cancer is a multifactorial disease and one of the leading causes of mortality worldwide. [1]. Efforts in combating cancer have been thwarted by the negative side effects and drug resistance associated with some of the available anticancer drugs [2]. Colon adenocarcinoma is a highly prevalent and deadly cancer that is often resistant to conventional therapies [3]. Therefore, the need for safer, more effective treatment options is non-negotiable. Natural products, particularly those derived from plants, offer promising therapeutic potential due to their rich chemical diversity and multi-targeted mechanisms of action [4,5]. Phytochemicals from medicinal plants have been shown to modulate key cellular processes that are frequently dysregulated in cancer, including cell cycle regulation, apoptosis, autophagy, and oxidative stress responses [6]. These bioactive compounds can induce programmed cell death and autophagy, and some act by altering reactive oxygen species (ROS) levels, leading to DNA damage and selective elimination of cancerous cells [7,8]. Apoptosis, a highly regulated form of programmed cell death, plays crucial roles in development, tissue homeostasis, and in eliminating damaged or potentially harmful cells under pathological conditions, thereby maintaining cellular homeostasis [9,10]. The pleiotropic effects associated with plant-derived phytochemicals have placed them as valuable candidates for the development of safer and more effective anticancer therapies.

Recently, we reported the anticancer mechanism of action of *Khaya grandifoliola*, which is commonly used in traditional medicine to modulate autophagy-induced apoptosis [11]. *Aristolochia ringens* may similarly modulate key oncogenic pathways, making it a potential source of novel anticancer agents. The root of *A. ringens* is traditionally used for its anti-inflammatory and analgesic properties by the Yoruba people in Southwestern Nigeria [12]. Studies have established the anticancer potency of the crude extract of the plant [13]; however, the underlying molecular mechanisms responsible for the therapeutic activity are still unknown. Despite the traditional medicinal use and confirmed anticancer activity of *A. ringens* [13], no prior study has investigated how the plant exerts these effects at the cellular level. In this study, we examined the effects of the methanolic root extract of *A. ringens* on colon adenocarcinoma cells, with a specific focus on its ability to induce apoptosis, alongside potential involvement of cell cycle arrest and mitochondria-mediated pathways. Understanding these mechanisms is essential for validating its therapeutic potential and supporting future anticancer drug development.

## 2. Results

### 2.1. A. ringens Crude Extract Posed Moderate Cytotoxicity Against Colon Adenocarcinoma Cells

**Figure 1:**
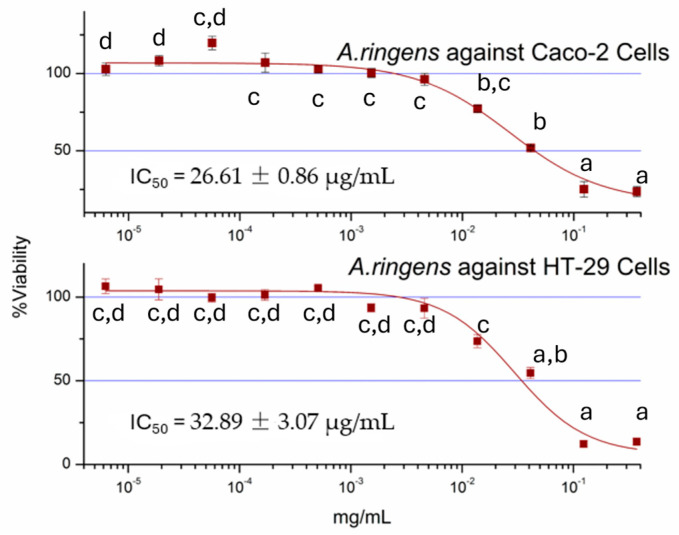
Percentage viability of Caco-2 and HT-29 cells following treatment with *A. ringens* extract. The %viability data are presented as mean ± standard deviation. ^a,b,c,d^ are the means within the same column, distinguished by different superscripts and exhibiting significant differences (*p* < 0.05).

The crude methanolic extract of *A. ringens* significantly reduced the viability of two human colon cancer cell lines, Caco-2 and HT-29, as determined by MTT assay. Figure 1 shows a dose-dependent cytotoxic effect observed in both cell lines following 72 h exposure, with IC_50_ values of 26.61 ± 0.86 µg/mL for Caco-2 and 32.89 ± 3.07 µg/mL for HT-29 (n = 3). The reduction in cell viability was statistically significant compared to the vehicular control (*p* < 0.05).

This bioactivity is consistent with typical bioactivity profiles observed for plant crude extracts, which often contain a mixture of bioactive and inactive constituents. Meanwhile, it is imperative to further perform bioactivity-guided fractionation, as the isolation of active secondary metabolites frequently yields fractions or pure compounds with enhanced potency.

### 2.2. Bioactivity-Guided Fractionation of A. ringens Crude Extract

**Figure 2:**
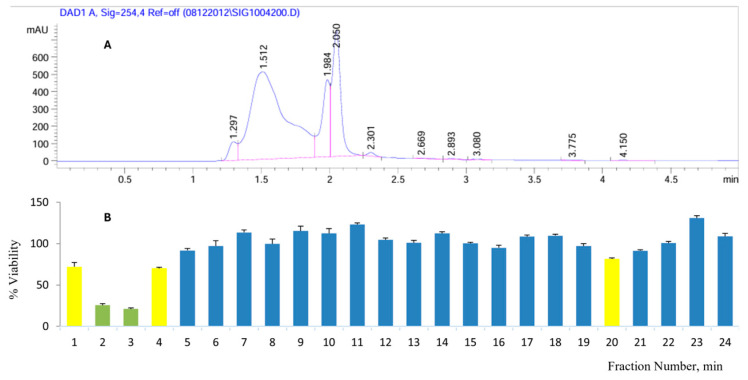
Bioactivity-guided fractionation of *A. ringens* extract. (**A**) Chromatograph obtained from HPLC fractionation. (**B**) Bioactivity potential of the fractions obtained from the HPLC separation. The bioactive fractions correspond to the observed peaks in the chromatograph.

The exploration of anticancer bioactive constituents in *A. ringens* through bioactivity-guided fractionation identified fractions F2 and F3 using semi-preparative HPLC with a C18 column (250 × 4.6 mm, 5 µm) and a water–acetonitrile gradient. Fractions were collected at a flow rate of 1 mL/min. Figure 2 presents the data from chromatographic separation and the subsequent bioactivity evaluation using the MTT assay. The chromatogram (Figure 2A), monitored at 254 nm, showed distinct peaks eluting between 1.4 and 2.7 min. A total of 24 fractions were collected and assessed for cytotoxicity against Caco-2 cells using the MTT assay. The results (Figure 2B) revealed that fractions F2 and F3, eluting at 2 and 3 min, respectively, significantly reduced Caco-2 cell viability after 72 h of treatment. Specifically, F2 reduced viability to 25.61% ± 3.1%, and F3 to 20.99% ± 2.8%, compared to the vehicle control (n = 3, *p* < 0.05). These findings suggest that the constituents responsible for the anticancer activity of the crude extract are concentrated within these two elution windows. The combined chromatographic and bioactivity data support the enrichment of active compounds in F2 and F3 and warrant further investigation.

### 2.3. Microscopic Characterization of Apoptotic Phenotypes Induced by A. ringens

**Figure 3:**
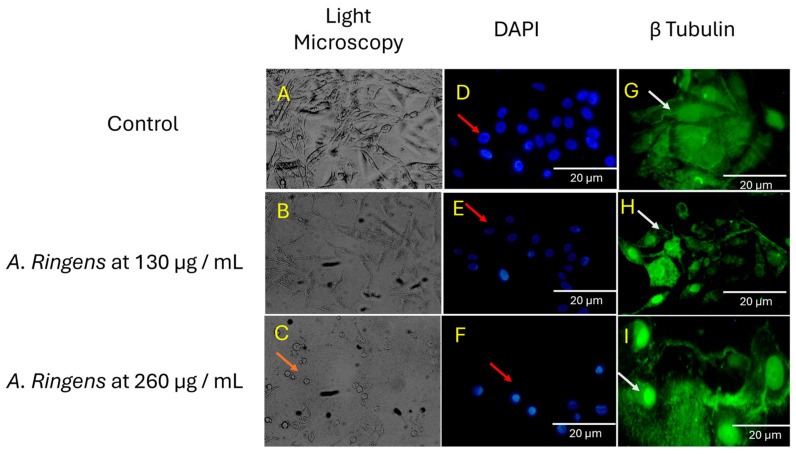
Representative photomicrographs showing the phenotypic effects of *A. ringens* extract on Caco-2 cells. (**A**–**C**) Light microscopy images (20×) demonstrate morphological changes, including cell rounding, detachment, and reduced confluence, consistent with cytotoxic and apoptotic responses. (**D**–**I**) Immunofluorescence images (40×) stained with β-tubulin (green) and DAPI (blue) reveal cytoskeletal disorganization and nuclear shrinkage in treated cells compared to the untreated control.

Mechanistic exploration of the effect of *A. ringens* on cytoskeletal organization and cellular morphology revealed features consistent with apoptosis. Figure 3 presents the dose-dependent effects of *A. ringens* on the cellular architecture of Caco-2 cells.

DAPI staining of the nuclei demonstrated morphological changes characteristic of apoptosis, including nuclear shrinkage and chromatin condensation, compared to the untreated controls. Quantification of nuclear shrinkage was performed using ImageJ (1.8.0-345) analysis alongside visual assessment. In vehicular control cells, approximately 24 nuclei were observed, with only 2–3 showing mild condensation, corresponding to 8–12.5% nuclear shrinkage. At 130 µg/mL of *A. ringens* extract, 7 out of 19 nuclei (37%) showed obvious signs of nuclear shrinkage. Notably, at 260 µg/mL, 6 out of 8 nuclei (75%) appeared condensed, brightly stained, and reduced in size. These findings demonstrate a dose-dependent increase in nuclear condensation, supporting the apoptotic potential of *A. ringens*.

To investigate the effect on the cytoskeleton, Caco-2 cells were stained with anti-tubulin antibody. Untreated cells displayed well-organized microtubule filaments radiating from the perinuclear region. In contrast, cells treated with 130 µg/mL extract show partial microtubule disruption and reduce filament density. At 260 µg/mL, severe cytoskeletal disintegration, increased cytoplasmic granularity, and apoptotic morphology, such as cell shrinkage and rounding, were observed. Although this was assessed qualitatively, the structural breakdown aligns with late-stage apoptotic features, suggesting that microtubule degradation may occur secondary to apoptosis.

### 2.4. A. ringens Induced Cell Cycle Arrest at G_1_ Phase

**Figure 4:**
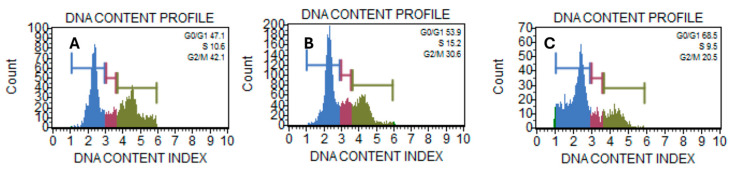
Cell cycle distribution of Caco-2 cells following treatment *with A. ringens* methanolic extract. (**A**) The distribution of Caco-2 cells treated with methanol as a vehicular control. (**B**,**C**) The distribution of Caco-2 cells treated with *A. ringens* at 130 µg/mL and 260 µg/mL, respectively.

Further mechanistic investigation into the cytotoxicity of *A. ringens* shows that the effect is associated with the modulation of the progression of cell cycle stages. Flow cytometric analysis via propidium iodide-stained Caco-2 cells reveals a dose-dependent increase and consistent decrease across the cell cycle stages. Table 1 shows significant phase shifts across treatment groups. In the G_0_/G_1_ phase, *A. ringens* at 260 µg/mL (67.2 ± 3.1%) significantly increased the cell population compared to control (42.6 ± 4.9%) and *A. ringens* at 130 µg/mL (52.4 ± 1.6%) with *p* value < 0.05. The S phase showed a significant rise at 130 µg/mL (15.8 ± 1.2%) and a drop at 260 µg/mL (6.4 ± 2.6%) with *p* < 0.05, suggesting a dose-dependent effect. G_2_/M phase exhibited a decreasing trend from control (35.8 ± 7.2%) to 260 µg/mL (23.9 ± 4.7%) but was not statistically significant. These findings indicate G_0_/G_1_ arrest at high doses and transient S phase accumulation at lower doses. Figure 4A–C shows the histogram representation of the DNA content of treated and untreated cells. The comparison of the DNA content between the treated sample and the control clearly showed a redistribution of the cell population. The observed G_1_ arrest could be an indication of a robust blockade at the G_1_ checkpoint. Corroborating the G_1_ arrest with the extract’s anticancer effect on cell viability may implicate an early mechanism contributing to downstream apoptosis induction. In addition, the phenotypic evidence from immunofluorescence studies, where treated cells exhibited nuclear shrinkage and disrupted microtubule network, is a hallmark of apoptotic stress and may be linked to cell cycle dysregulation.

### 2.5. A. ringens Induced Cell Death via Apoptosis Induction

**Figure 5:**
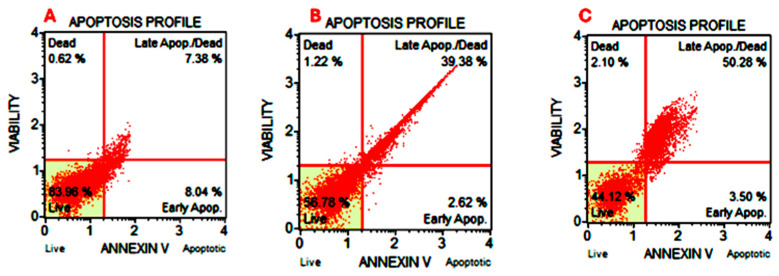
Representative flow cytometry quadrants showing apoptosis induction in Caco-2 cells after treatment with *A. ringens*: (**A**) vehicular control, (**B**) *A. ringens* treated at 130 µg/mL, and (**C**) *A. ringens* treated at 260 µg/mL.

To confirm the apoptotic induction of *A. ringens* crude extract on Caco-2 cells, Annexin V-FITC/PI staining was performed, and the percentages of live, early apoptotic, late apoptotic, and necrotic cells were quantified. Figure 5 displays representative flow cytometry quadrants showing cell distribution among live, early apoptotic, late apoptotic, and necrotic populations. Treatment with *A. ringens* extract resulted in a dose-dependent decrease in the live cell population and a corresponding increase in late apoptosis. At 130 µg/mL concentration, the live cell population significantly decreased to 56.12 ± 0.87%, and further dropped to 43.64 ± 1.45% at 260 µg/mL, compared to 86.23 ± 1.99% in the control group (*p* < 0.05). Early apoptotic cells remained unchanged across all groups (no statistical significance). However, late apoptotic cells significantly increased from 7.57 ± 0.36% in the control to 39.05 ± 1.61% and 50.03 ± 0.50% at 130 µg/mL and 130 µg/mL, respectively (*p* < 0.05). These findings confirm that *A. ringens* induced a strong, dose-dependent pro-apoptotic effect in colon cancer cells, primarily through the induction of late-stage apoptosis.

### 2.6. A. ringens Disrupts Mitochondrial Membrane Potential

**Figure 6:**
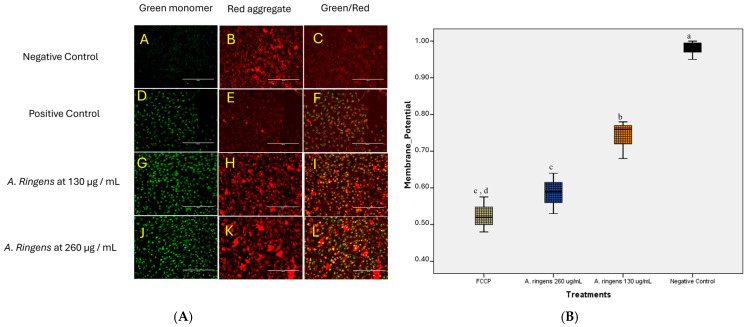
Loss of mitochondrial membrane potential in *A. ringens-treated* Caco-2 Cells. (**A**) Photomicrographs showing swift changes in the red and green signals after treatment with *A. ringens.* (**A**–**C**) Negative control (untreated cells) showing predominantly red fluorescence, indicative of intact mitochondrial membrane potential. (**D**–**F**) Positive control (FCCP-treated cells) showing loss of ΔΨm, with strong green fluorescence. (**G**–**I**) Cells treated with A. ringens at 130 µg/mL showing mixed red/green signals, indicating partial mitochondrial depolarization. (**J**–**L**) Cells treated with A. ringens at 260 µg/mL showing predominant green fluorescence, indicating extensive mitochondrial depolarization. Scale bar = 200 µm. (**B**) Box plot displaying the relative membrane potential quantification of red/green JC-1 signals ratio. The intensity data are presented as mean ± standard deviation. a,b,c,d are the means within the same column, distinguished by different superscripts and exhibiting significant differences (*p* < 0.05).

Treatment of Caco-2 cells with *A. ringens* extract resulted in a dose-dependent disruption of mitochondrial membrane potential, as assessed by JC-1 staining (Figure 6A). In vehicle control (methanol-treated cells), strong red fluorescence indicative of polarized mitochondria was observed. In contrast, cells treated with FCCP (carbonyl cyanide-p-trifluoromethoxyphenylhydrazone, a known mitochondrial uncoupler used as positive control) (positive control) and *A. ringens* extract (130 and 260 µg/mL) showed increased green fluorescence, reflecting mitochondrial depolarization.

Quantitative analysis of red/green fluorescence ratios demonstrated a dose-dependent decrease in mitochondrial membrane potential following *A. ringens* treatment (Figure 6B). The negative control (vehicle-treated) cells maintained a high membrane potential (mean ratio ≈ 0.96), which was significantly higher than all other treatment groups (n = 3, *p* < 0.05). Cells treated with 130 µg/mL *A. ringens* showed a moderate reduction in mitochondrial potential (0.74 ± 0.05), while those treated with 260 µg/mL exhibited a further decrease (0.58 ± 0.06). FCCP-treated cells, used as a positive control for mitochondrial depolarization, showed the lowest potential (0.50 ± 0.09). Statistical analysis revealed that FCCP and 260 µg/mL treatments were not significantly different from each other (*p* > 0.05), but both differed significantly from the 130 µg/mL group and control (*p* < 0.05). These findings indicate that *A. ringens* induces mitochondrial dysfunction in Caco-2 cells, supporting its role in apoptosis induction.

## 3. Discussion

The continued reliance on medicinal plants, particularly in developing regions, highlights the urgent need for mechanistic validation of their therapeutic claims. Despite their widespread use, many ethnomedicinal plants lack scientific evidence to support both their efficacy and safety in humans [14,15]. In this study, we provide mechanistic insight into the anticancer activity of the methanolic root extract of *A. ringens* in human colorectal carcinoma. The extract exhibited moderate cytotoxicity in both Caco-2 and HT-29 colon cancer cell lines, with IC_50_ values below 30 µg/mL, i.e., within the pharmacologically active range for crude plant extracts [16]. Our findings demonstrate, for the first time, that *A. ringens* induced G_1_ phase cell cycle arrest and mitochondria-mediated apoptosis, thereby offering a novel therapeutic angle against drug-resistant colorectal cancer. Given the burden of colon adenocarcinoma and its resistance to conventional chemotherapy [3], these findings contribute valuable knowledge toward the bioprospecting of new anticancer agents from plant sources.

Mechanistically, treatment of Caco-2 cells with *A. ringens* extract revealed hallmark features of apoptosis, including cell shrinkage, nuclear condensation, loss of adhesion, and β-tubulin degradation. These morphological changes indicate activation of programmed cell death pathways [17]. The observed degradation of tubulin suggests impairment of microtubule-dependent functions such as intracellular transport, centrosome stability, and mitotic progression. Disruption of microtubule dynamics can activate stress response pathways, notably the p53 signaling cascade, leading to upregulation of cyclin-dependent kinase inhibitors like p21 and subsequent G_1_ phase cell cycle arrest. Additionally, microtubule destabilization can compromise the mitochondrial network, triggering mitochondrial dysfunction, loss of membrane potential (ΔΨm), and release of apoptogenic factors such as cytochrome C, i.e., hallmarks of intrinsic apoptosis [18,19].

Preliminary GC–MS profiling of the bioactive fractions indicated the possible presence of steroidal and triterpenoid derivatives. For example, a steroidal compound with similarity to estra-5(10)-en-3-one-17-ol acetate was annotated, which could, in principle, influence tubulin dynamics in a manner reminiscent of certain microtubule-targeting steroids such as taccalonolides [20]. Likewise, a triterpenoid with structural similarity to ginsenol was detected, sharing features with ginsenosides Rg3 and Rh2, which have been reported to induce mitochondrial depolarization, ROS accumulation, and caspase activation [21,22]. These annotations are tentative, based solely on spectral matching, and the compounds were neither isolated nor functionally validated in the present study. Nevertheless, their predicted presence is consistent with the observed G1 arrest, mitochondrial perturbation, and apoptotic phenotype, supporting our proposed model in which *A. ringens* disrupts cytoskeletal integrity and mitochondrial homeostasis, converging on intrinsic apoptosis as a plausible anticancer mechanism.

Despite the promising mechanistic insights provided in this study, the arising limitations warrant further examination. The use of a crude methanolic extract introduces compositional complexity, limiting the ability to attribute the observed effects to specific bioactive constituents. Although the preliminary GC–MS analysis tentatively annotated compounds such as estra-5(10)-en-3-one-17-ol acetate and ginsenol, their mechanistic involvement remains hypothetical and requires validation through isolation, structural characterization, and targeted biological assays. Furthermore, the in vitro findings, while mechanistically informative, do not account for pharmacokinetic behavior, systemic toxicity, or therapeutic efficacy in vivo. Notably, the potential presence of aristolochic acids (compounds known for nephrotoxicity and carcinogenicity in some *Aristolochia* spp.) raises important safety concerns [23,24] that must be addressed prior to clinical consideration. Future investigations should therefore prioritize bioactivity-guided fractionation, identification of the active principle(s), toxicological evaluation, and in vivo efficacy studies to substantiate the therapeutic relevance and safety profile of *A. ringens* as a candidate anticancer agent.

## 4. Materials and Methods

### 4.1. Plant Extraction

The root of *A. ringens* was obtained in Iwo Township, Osun State, Nigeria. The plant specimen was deposited at the Herbarium of the University of Ibadan and assigned with Voucher No. UIH-22018, accessible for reference. The plant sample was allowed to air-dry at room temperature in a well-ventilated area. Thereafter, the sample was crushed into a fine powder before maceration in methanol to obtain the crude extract; 20 g of the fine powder of the plant part was macerated in 200 mL, 95% *v*/*v* methanol obtained from Sigma, St. Louis, MO, USA, and left on a shaker (150 rpm for 24 h at room temperature). The mixture was filtered after 24 h, and the residue was re-macerated in methanol for further extraction under the same conditions. The two extracts were later pooled together and concentrated using a Büchi Rotavapor® R-300 (Büchi Labortechnik AG, Flawil, Switzerland) at 40 °C. The concentrate was dissolved in methanol as 10 mg/mL and stored at −20 °C until further use.

### 4.2. Cell Viability Assay

The cytotoxic effects of the crude and the fractions obtained from the HPLC were assessed via MTT (3-(4,5-dimethylthiazol-2-yl)-2,5-diphenyltetrazolium bromide) assay, with slight modification to the Elnakady protocol [25]. Briefly, 120 µL of 50,000 cells per mL Caco-2 and HT29 cells (ATCC collection: Caco-2: HTB-37 and HT-29: HTB-38) were seeded in 96-well plates and then treated with 60 µL from the serially diluted treatments. The plates were treated after 72 h incubation in a CO_2_ incubator with 20 µL MTT salt (20 mg/mL). Two hours after MTT incubation, the addition of the MTT salt produced formazan crystals. Thereafter, the solution was gently aspirated by a vacuum aspirator, without disrupting the formazan crystal. The formazan was dissolved in 200 µL of isopropanol and incubated for an additional 10 min. The plates were then put on a shaker for 10 min, and the optical densities were read using Biochrom® Anthos 2010 microplate reader (Biochrom Ltd., Cambridge, UK) at 595 nm. The viability test was performed in three independent experiments in triplicate. IC_50_ values of the crude extract were obtained using Origin 6.1 software (Origin Lab Corporation, Northampton, MA, USA).

### 4.3. Cellular Phenotyping Assays

Caco-2 cells were cultured on coverslips, and treatment was conducted at around 75% confluence. Crude extract of *A. ringens* at 5× and 10× IC_50_ values (corresponding to 130 µg/mL and 260 µg/mL, respectively) was added to the cell; 0.1% (*v*/*v*) of methanol in culture media was used as a vehicular control. After 24 h, the cells on the cover slips were fixed with 1:1 acetone/methanol. Fixed cells were then stained with mouse anti-tubulin antibody, and Alexa 488-conjugated secondary antibodies (goat anti-mouse) were used in conjunction with the primary antibodies. 4-6-Diamidino-2-phenylindole (DAPI) was used to counterstain the nucleus [26]. Slides were prepared, and antifade was applied to reduce photo-bleaching before the scoring with the Olympus fluorescence microscope.

### 4.4. Cell Cycle Fraction Analysis

The distribution of cells across the cell cycle phases was analyzed by staining the treated Caco-2 cells with crude *A. ringens* at 130 µg/mL and 260 µg/mL, and methanol was used as a vehicular control at 0.1% (*v*/*v*) in culture media. Cells were harvested by centrifugation and fixed in 1:1 ice-cold PBS and ethanol 24 h post-treatment. Thereafter, the cells were washed with PBS, and then treated with 0.1% *w*/*v* saponin, RNAse, and the nuclei were stained with propidium iodide (30 min at 37 °C) [27]. Cell cycle fractions were analyzed with a Muse flow cytometer (Sigma, St. Louis, MO, USA).

### 4.5. Apoptosis Detection Assay

The induction of apoptosis by *A. ringens* was measured via Annexin V FITS apoptosis detection kit from Abcam (ab14085; Abcam plc, Cambridge, UK). Following the kit protocol, apoptosis was induced in Caco-2 cells by treatment with the plant crude extract at concentration levels of 130 µg/mL and 260 µg/mL; 0.1% (*v*/*v*) of methanol in culture media was used as a vehicular control. Treated cells were harvested via centrifugation 24 h after incubation in a 5% CO_2_ incubator at 37 °C. Harvested cells were carefully washed with PBS and stained with fluorescein-conjugated Annexin V FITC and propidium iodide for 30 min at room temperature in the dark; 500 μL annexin V binding buffer was added to the cells, and percentage apoptosis was measured using a flow cytometer FACS Muse cell analyzer (Sigma, St. Louis, MO, USA).

### 4.6. Mitochondria Membrane Potential (Δψm) Assay

The effect of the crude *A. ringens* on mitochondrial membrane potential was evaluated using the JC-1 Mitochondrial Membrane Potential Assay Kit (ab113850, Abcam plc, Cambridge, UK). The kit protocol was adapted to modification for live fluorescence imaging. Caco-2 cells were seeded on sterile glass coverslips in a 4-well culture dish at a density of 1.0 × 10^5^ cells per well and incubated at 37 °C with 5% CO_2_ for 24 h. Cells were then treated with *A. ringens* crude extract at concentrations of 130 µg/mL and 260 µg/mL for 24 h. A subset of wells was treated with 20 µM FCCP (carbonyl cyanide-p-trifluoromethoxyphenylhydrazone) for 1 h prior to staining as a positive control for mitochondrial depolarization, and another well for vehicular control with methanol at 0.1% (*v*/*v*) in culture media.

After treatments, the culture media were removed and washed ×2 with supplemented buffer before a pre-warmed JC-1 working solution (2 µM in assay buffer). The cells were incubated for 25 min at 5% CO_2_, 37 °C in the dark. Following the staining procedure, cells were washed twice with JC-1 assay buffer supplemented with 25 mM HEPES to stabilize pH during imaging, as live-cell imaging was conducted outside of a CO_2_ incubator. Images were acquired immediately using an EVOS FL fluorescence microscope (Thermo Fisher Scientific, Waltham, MA, USA). JC-1 monomers (indicative of depolarized mitochondria) were visualized using the green (FITC) channel (excitation/emission ~488/530 nm), while JC-1 aggregates (indicative of polarized mitochondria) were visualized using the red (TRITC) channel (excitation/emission ~540/590 nm). Exposure settings were kept constant across samples. Images were acquired promptly to minimize photo-bleaching and dye leakage.

Qualitative assessment of mitochondrial polarization was based on the red-to-green fluorescence pattern. Quantitative analysis was performed using ImageJ (1.8.0-345)software to measure the mean fluorescence intensity of red and green signals and to calculate the red/green fluorescence ratio as a proxy for ΔΨm status.

### 4.7. Bioactivity-Guided Fractionation and Phytochemical Profiling

#### 4.7.1. Preliminary HPLC Profiling

The crude methanolic extract of *A. ringens* root was initially subjected to reverse-phase HPLC analysis using an Agilent Technologies 1290 Infinity system (Agilent Inc., Palo Alto, CA, USA) to obtain a chemical profile and guide fractionation. Separation was performed on a ZORBAX RX-C18 analytical column (4.6 × 150 mm) using a gradient elution of acetonitrile and water containing 0.1% formic acid, with UV detection set at 254 nm. An injection volume of 50 µL per run at a concentration of 10 mg/mL was used. To obtain all 24 fractions, the HPLC run was repeated multiple times, and eluates were collected in 1 min intervals across the entire run time. This time-based fractionation approach ensured compatibility with the column’s capacity, while generating sufficient material for downstream bioactivity-guided screening. The collected fractions were subjected to preliminary cytotoxicity evaluation using the MTT assay.

#### 4.7.2. GC–MS Identification

A preliminary GC–MS analysis was conducted to explore the semi-volatile components of the bioactive fraction. Bioactive fractions obtained from HPLC were pooled, dried under a rotary evaporator, and reconstituted in methanol prior to analysis; 2 µL was injected into an Agilent 5977B Gas Chromatograph (Agilent Technologies, Santa Clara, CA, USA) coupled with a Mass Selective Detector (GC/MSD). Gas chromatographic separation was achieved using an HP-5MS Ultra Inert (UI) capillary column (30 m length × 0.25 mm internal diameter × 0.25 µm film thickness). Helium was used as the carrier gas at a constant ~1 mL/min flow rate. The oven temperature program was set at an initial temperature of 80 °C, which was held for 2 min, to enable sample focusing and initial separation. Subsequently, a programmed temperature ramps up to 300 °C. The total running time was 52 min to ensure complete elution of all analytes. Mass spectrometric detection was performed using electron ionization (EI) mode at 70 eV, with the ion source temperature maintained at 230 °C and the quadrupole temperature at 150 °C. The solvent delay time was set at 7 min to prevent solvent peak interference during ion monitoring. The mass spectrometer scanned over a mass-to-charge (*m*/*z*) range, suitable for the expected analytes, *m*/*z* 40–550.

Data acquisition and processing were performed using Agilent Mass Hunter software 10.0. Compound identification was based on a comparison of acquired mass spectra with those in the National Institute of Standards and Technology (NIST 14.L) mass spectral library, considering matching scores, retention indices, and retention times. We acknowledge the limitations of GC–MS in profiling methanol-based root extracts; therefore, the results are presented in the Supplementary Data (Table S1, Figure S1) as tentative annotations to support future dereplication efforts.

### 4.8. Statistical Analysis

The data generated from this study were statistically analyzed using SPSS (version 20). One-way analysis of variance (ANOVA) was performed to separate the means and significance level determination at (*p* < 0.05). ^a,b,c,d^ mean significant differences.

## 5. Conclusions

This study demonstrates that the methanolic root extract of *A. ringens* exerts significant anticancer effects against Caco-2 human colorectal adenocarcinoma cells by inducing G_1_ phase cell cycle arrest and mitochondrial-mediated apoptosis. The dose-dependent reduction in cell viability, morphological evidence, and mitochondrial membrane depolarization evidenced these effects. However, the findings have limitations, including the use of a crude extract, which contains a complex mixture of compounds, the absence of in vivo validation, and molecular target confirmation. As such, the specific bioactive constituents and their precise molecular targets remain to be elucidated. Future studies should focus on the isolation and structural characterization of the anticancer bioactive compounds from *A. ringens*, the validation of molecular targets such as caspases, p21, and cytochrome C using Western blot or immunofluorescence analysis, and in vivo evaluation of the extract’s therapeutic efficacy and toxicity profile. These steps are essential for advancing *A. ringens* toward preclinical development as a potential anticancer agent.

## Figures and Tables

**Table 1:** Cell cycle phase distribution after *A. ringens* treatments.

Cell Cycle Phase	Control (% ± SD)	130 µg/mL (% ± SD)	260 µg/mL (% ± SD)
G_0_/G_1_	42.6 ± 4.9 ^a^	52.4 ± 1.6 ^b^	67.2 ± 3.1 ^c^
S	11.9 ± 2.4 ^a^	15.8 ± 1.2 ^b^	6.4 ± 2.6 ^c^
G_2_/M	35.8 ± 7.2 ^a^	31.3 ± 2.8 ^a^	23.9 ± 4.7 ^a^

Note: Values are presented as mean ± SD; n = 3; ^a,b,c^ statistical groups within each row. Groups that do not share the same letter are significantly different (Tukey’s HSD, *p* < 0.05).

## Data Availability

Data are as contained in the article and supplementary materials.

## Supplementary Materials

The following supporting information can be downloaded at https://www.mdpi.com/article/10.3390/ph18091250/s1, Figure S1: GCMS chromatograph of the bioactive fractions of *A. ringens*; Table S1: GCMS metabolite Profiling of tentative anticancer phytochemicals present in *A. ringens*; and Spectral Information. References [28,29,30,31,32,33] are cited in the supplementary materials.

## Abbreviations

The following abbreviations are used in this manuscript:

MTT	(4,5-Dimethylthiazol-2-yl)-2,5-diphenyltetrazolium bromide
FCCP	Carbonyl cyanide-p-trifluoromethoxyphenylhydrazone
HPLC	High-performance liquid chromatography
GC–MS	Gas chromatography–mass spectrometry
ΔΨm	Electrochemical gradient across the inner mitochondrial membrane
