# Supplementary material for: Bioactivity-Guided Fractionation and Mechanistic Insights into Aristolochia ringens Root Extract-Induced G1 Phase Arrest and Mitochondria-Mediated Apoptosis in Human Colon Adenocarcinoma Cells
Source: Pharmaceuticals (Basel). 2025 Aug 23;18(9):1250. doi: 10.3390/ph18091250 (PMC12472686; doi:10.3390/ph18091250)
Supplement: Supplementary file 1 [file pharmaceuticals-18-01250-s001.zip › pharmaceuticals-3761029-supplementary.pdf]

Article

Bioactivity-Guided Fractionation and Mechanistic Insights into *Aristolochia ringens* Root Extract-Induced G_1 Phase Arrest and Mitochondria-Mediated Apoptosis in Human Colon adenocarcinoma Cells.

Saheed O. Anifowose [1], Abdalrhaman M. Salih [2], Musa K. Oladejo [1], Ahmad Rady [1], Mobarak S. Al Mosallam [3], Hasan A Aljohi [4], Mansour I. Almansour [1], Saad Hussin Alkahtani [1], Ibrahim. O. Alanazi [3*] and Badr A. Al-Dahmash [1 *]

[1] Zoology Department, College of Science, King Saud University, P.O. Box 2455, Riyadh 11451, Saudi Arabia
[2] Botany and Microbiology Department, College of Science, King Saud University, P.O. Box 2455, Riyadh 11451, Saudi Arabia
[3] Healthy Aging Research Institute, Health Sector, King Abdulaziz City for Science and Technology, Riyadh 11442, Saudi Arabia
[4] Applied Genetics Technology Institute, King Abdulaziz City for Science and Technology, Riyadh 11442, Saudi Arabia
* Correspondence: researchoutput_baldhmash@hotmail.com; ialenazi@kacst.gov.sa

Supplementary Data

Results

GCMS Metabolite Profiling of A. ringens Bioactive Fractions

To gain preliminary insight into the chemical composition of the bioactive fractions F2 and F3, GC-MS analysis was performed. The resulting chromatogram showed late-eluting peaks, consistent with non-volatile or thermally stable compounds (Supplementary Table S1 and Figure S1). Based on spectral similarity with entries in the NIST library, a range of compound classes were tentatively annotated, including alkaloids, sesquiterpenes, diterpenes, and steroid-like structures.
Notably, one peak showed high similarity to estra-5(10)-en-3-one-17-ol acetate, a steroidal derivative previously associated with antiproliferative activity, while another peak aligned with 1,5-cyclodecadiene derivatives, compounds within the sesquiterpene class known to possess cytotoxic or pro-apoptotic properties. However, these annotations remain putative and are presented here solely for hypothesis-generating purposes.

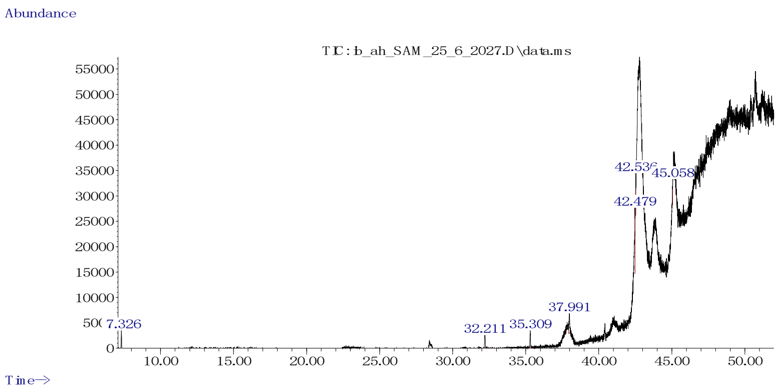

Supplementary Figure S1. GCMS chromatograph of the bioactive fractions of *A. ringens*

Supplementary Table S1. GCMS metabolite Profiling of tentative anticancer phytochemicals present in *A. ringens*

RT	Putative Compound	M. Formula	MW	Area %	Library RI	Estimated RI	Δ RI (Library – Estimated)	Notes
37.991	Esra-5(10)-en-3-one-17-ol, acetate	$C_{20}H_{28}O_3$	316	15.27	2234	2190.00	+44.0	NIST Library RI used in original output; estimated RI computed with van den Dool & Kratz from a reference HP-5MS alkane ladder (provisional). No HP-5 literature RI found.
42.479	Germacrene B (1,5-Cyclodecadiene…, (E,E))	$C_{15}H_{24}$	204	36.3	1603	1566.67	+36.33	Literature HP-5/DB-5 RI ≈ 1554 (see SI references); estimated RI is close but shows a moderate discrepancy (Δ ≈ 12.7 vs literature 1554).
42.536	2-Methyl-6-(5-methyl-2-thiazolin-2-ylamino)pyridine	$C_{10}H_{13}N_3S$	207	14.36	1790	1573.33	+216.67	NIST Library RI; estimated RI differs substantially (ID unreliable without experimental RI or standards).
45.05	Ginsenol	$C_{15}H_{26}O$	222	8.22	1432	1648.65	-216.65	NIST Library RI used in original output; literature RI found on polar columns (e.g., Supelcowax ≈ 2155), not HP-5. Therefore, not directly comparable. Estimated RI here is provisional.

Spectral Information

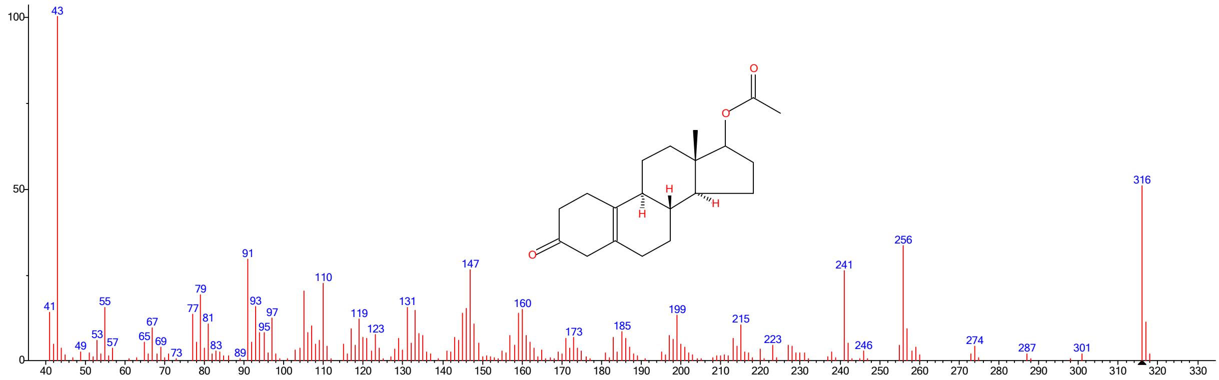

(mainlib) Estra-5(10)-en-3-one-17-ol, acetate

Name: Estra-5(10)-en-3-one-17-ol, acetate
Formula: $C_{20}H_{28}O_3$
MW: 316 Exact Mass: 316.203844 CAS#: 19906-32-2 NIST#: 124060 ID#: 14638 DB: mainlib
Other DBs: None
Contributor: H. Fales, LC, NHLBI, NIH, Bethesda, MD 20892
InChIKey: JKSYUQKNAOZGAO-QDSUGMFFSA-N Non-stereo
10 largest peaks:
43 999 | 316 506 | 256 334 | 91 293 | 147 262 |
241 259 | 110 223 | 105 200 | 79 191 | 93 156 |
Synonyms:
1.Estr-5(10)-en-17-ol-3-one, (17β-, acetate
2.Androst-5(10)-en-17.beta-ol-3-one acetate
3.3-Oxoestr-5(10)-en-17-yl acetate #

Estimated non-polar retention index (n-alkane scale):
Value: 2234 iu
Confidence interval (Low reliability): 174(50%) 752(95%) iu

5.

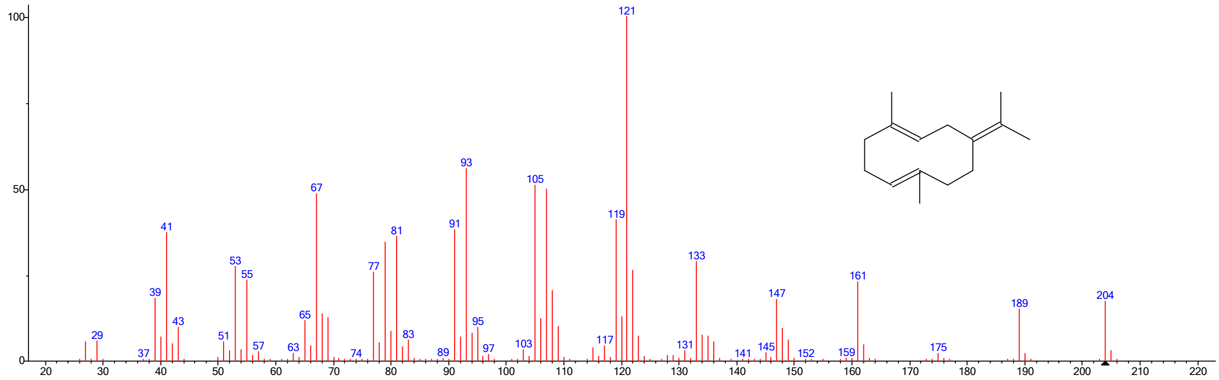

(mainlib) 1,5-Cyclodecadiene, 1,5-dimethyl-8-(1-methylethylidene)-, (E,E)-

Name: 1,5-Cyclodecadiene, 1,5-dimethyl-8-(1-methylethylidene)-, (E,E)-
Formula: $C_{15}H_{24}$
MW: 204 Exact Mass: 204.1878 CAS#: 15423-57-1 NIST#: 384129 ID#: 102819 DB: mainlib
Other DBs: None
Contributor: NIST Mass Spectrometry Data Center, 2010
InChIKey: GXEGJTGWYVZSNR-LBJJKJHXSA-N Non-stereo

10 largest peaks:

121 999 |	93 557 |	105 511 |	107 498 |	67 485 |
119 409 |	91 382 |	41 373 |	81 362 |	79 345 |

Synonyms:

1.Germacrene B
2.Germacra-1(10),4,7(11)-triene
3.Germacra-1(10),4,7(11)-triene, (E,E)-
4.1,5-Dimethyl-8-(1-methylethylidene)-1,5-cyclodecadiene #
5.(1E,5E)-1,5-Dimethyl-8-(propan-2-ylidene)cyclodeca-1,5-diene
6.1,5-Cyclodecadiene, 1,5-dimethyl-8-(1-methylethylidene)-, (1E,5E)-

Experimental RI median±deviation (#data)
Semi-standard non-polar: 1557±3 (233)
Standard non-polar: 1550±7 (91)
Polar: 1819±19 (67)
Estimated non-polar retention index (n-alkane scale):
Value: 1603 iu
Confidence interval (Hydrocarbons): 39(50%) 167(95%) iu

Retention index.
1. Value: 1554 iu
Column Type: Capillary
Column Class: Standard non-polar
Active Phase: RTX-1
Column Length: 60 m
Carrier Gas: He
Column Diameter: 0.22 mm
Phase Thickness: 0.25 um
Data Type: Linear RI
Program Type: Ramp
Start T: 60 C
End T: 230 C
Heat Rate: 2 K/min
End Time: 35 min
Source: Paolini J.; Costa J.; Bernardini A.F., **Analysis of the essential oil from the roots of Eupatorium cannabinum subsp corsicum (L.) by GC, GC-MS and C-13-NMR**, *Phytochem. Anal.*, *18*, **2007**, 235-244.

2. Value: 1534 iu
Column Type: Capillary
Column Class: Standard non-polar
Active Phase: SPB-1
Column Length: 30 m
Carrier Gas: He
Column Diameter: 0.25 mm
Phase Thickness: 0.25 um
Data Type: Linear RI
Program Type: Ramp
Start T: 50 C
End T: 250 C
Heat Rate: 5 K/min
Start Time: 3 min
End Time: 15 min
Source: Radulovic, N.; Mananjarasoa, E.; Harinantenaina, L.; Yoshinori, A., **Essential oil composition of four Croton species from Madagascar and their chemotaxonomy**, *Biochem. Syst. Ecol.*, *34*, **2006**, 648-653.

6. 1000159-38-5 NOT found and second one chosen 339352-50-0

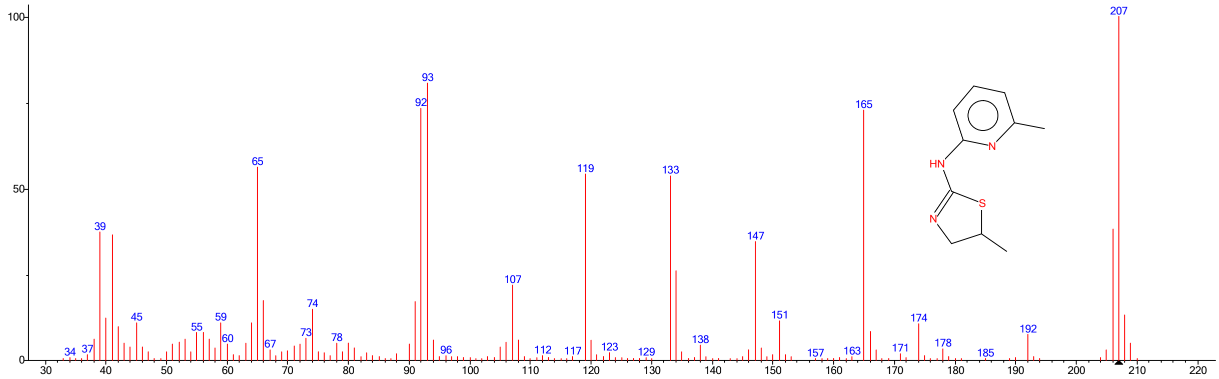

(mainlib) 2-Methyl-6-(5-methyl-2-thiazolin-2-ylamino)pyridine

Name: 2-Methyl-6-(5-methyl-2-thiazolin-2-ylamino)pyridine
Formula: $C_{10}H_{13}N_3S$
MW: 207 Exact Mass: 207.083018 CAS#: 339352-50-0 NIST#: 225393 ID#: 188677 DB: mainlib
Other DBs: None
Contributor: Chemical Concepts
InChIKey: CMHNKMRKUZJKCO-UHFFFAOYSA-N Non-stereo
10 largest peaks:
207 999 | 93 805 | 92 733 | 165 726 | 65 561 |
119 542 | 133 535 | 206 380 | 39 373 | 41 363 |
Synonyms:
1.6-Methyl-N-(5-methyl-4,5-dihydro-1,3-thiazol-2-yl)-2-pyridinamine #

Estimated non-polar retention index (n-alkane scale):
Value: 1790 iu
Confidence interval (Diverse functional groups): 89(50%) 382(95%) iu

7. 117591-80-7

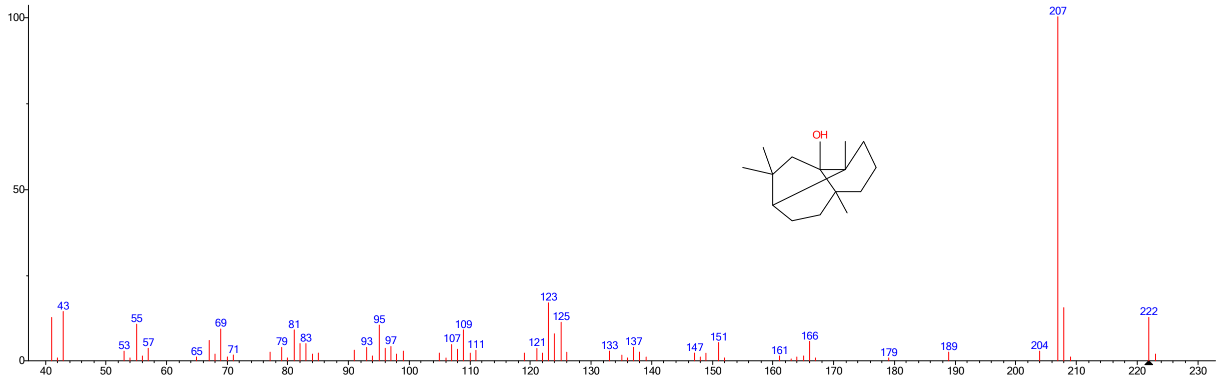

(mainlib) Ginsenol

Name: Ginsenol
Formula: $C_{15}H_{26}O$
MW: 222 Exact Mass: 222.198365 CAS#: 117591-80-7 NIST#: 140227 ID#: 188727 DB: mainlib
Other DBs: None
Contributor: B. Derendyaev, Novosibirsk Institute of Organic Chemistry
InChIKey: QOXUIQMPPDIDGM-UHFFFAOYSA-N Non-stereo
10 largest peaks:
207 999 | 123 167 | 208 152 | 43 142 | 222 125 |
41 124 | 125 111 | 55 104 | 95 102 | 69 90 |
Synonyms:
1.3,3,7,11-Tetramethyltricyclo[5.4.0.0(4,11)]undecan-1-ol

Estimated non-polar retention index (n-alkane scale):
Value: 1432 iu
Confidence interval (Low reliability): 174(50%) 752(95%) iu

References

1. Adams, R. P. Identification of Essential Oil Components by Gas Chromatography/Mass Spectrometry, 4th ed.; Allured Publishing Corporation: Carol Stream, IL, USA, 2007.

   (Common source of HP-5 and DB-5 RI values for terpenoids and hydrocarbons; contains example n-alkane ladders.)

2. Van den Dool, H.; Kratz, P.D. A generalization of the retention index system including linear temperature programmed gas–liquid partition chromatography. Journal of Chromatography A 1963, 11, 463–471. https://doi.org/10.1016/S0021-9673(01)80947-X

(Original equation for calculating linear retention indices from n-alkane reference series.)

3. NIST Chemistry WebBook; NIST Standard Reference Database Number 69; National Institute of Standards and Technology: Gaithersburg, MD, USA.

   https://webbook.nist.gov/chemistry/ (Database of experimental RI values for various columns; HP-5 and DB-5 data used for literature comparisons.)

4. Shellie, R.; Mondello, L.; Marriott, P.; Dugo, G. Characterisation of lavender essential oils by using gas chromatography–mass spectrometry with correlation of linear retention indices and comparison with comprehensive two-dimensional gas chromatography. *Journal of Chromatography A* **2002**, *970*, 225-234.

   (Reports HP-5MS RI for Germacrene B around 1554 in Artemisia and other plant essential oils.)
